# Comparison of clinical and radiological outcomes after three different surgical treatments for resistant calcifying tendinitis of the shoulder: a short-term randomized controlled trial

**DOI:** 10.1186/s13018-022-03373-1

**Published:** 2022-11-05

**Authors:** Freek Verstraelen, Martijn Schotanus, Steffie Klemann-Harings, Okke Lambers Heerspink, Edwin Jansen

**Affiliations:** 1 Department of Orthopedics, Zuyderland MC, Dr. H. van der Hoffplein 1, 6162 BG Sittard, Limburg The Netherlands; 2 Faculty of Health, Medicine and Life Science, School of Care and Public Health Research Institute, Maastricht University, Universiteitssingel 40, 6229 ER Maastricht, Limburg The Netherlands; 3 Department of Orthopedics, Viecuri MC, Tegelseweg 210, 5912 BL Venlo, Limburg The Netherlands

**Keywords:** Calcifying tendinitis, Shoulder, Debridement, Subacromial decompression, Surgical treatment

## Abstract

**Background:**

A preferable surgical treatment for patients with conservative therapy-resistant calcifying tendinitis of the shoulder is still a matter of debate. Therefore, the purpose of this study was to evaluate and compare short-term clinical and radiological results of three surgical treatment options for these patients.

**Methods:**

A multicenter randomized trial was conducted. Sixty-nine patients were randomly assigned to receive 1. subacromial decompression (Group SAD), 2. debridement of calcifications (Group D), or 3. debridement of calcifications with SAD (Group D + SAD). Stringent inclusion and exclusion criteria were used. The primary outcome was an improvement in VAS for pain (pVAS) 6 months postoperatively. Secondary outcomes were an improvement in pVAS 6 weeks postoperatively, functional outcomes (CMS, DASH, ASES), radiological outcome, additional treatments, and complications.

**Results:**

The improvement in pVAS was significant in all groups (*p* < 0.001) and did not differ between the groups after 6 months. Six weeks postoperatively, the improvement in pVAS was significantly (*p* = 0.03) less in Group SAD compared to Group D + SAD (16.5 mm, SD 19.3 mm vs 33.1 mm, SD 19.7 mm, respectively). The mean size of calcifications decreased significantly in all groups (*p* < 0.0001). In Group SAD, the size of the calcifications decreased less (*p* = 0.04) compared to Group D and Group D + SAD after 6 weeks. Group SAD received more additional treatments (*p* = 0.003) compared to Group D + SAD (9 vs 1), which were mainly subacromial cortisone injections.

**Conclusions:**

All patient groups showed significant pain relief and an improvement in shoulder function 6 months after surgery. However, patients in Group SAD showed inferior pain relief and less improvement in DASH score after 6 weeks. Furthermore, this group required more postoperative additional treatments. No significant differences in clinical and radiological outcomes were observed between patients in Group D compared to Group D + SAD. Therefore, an arthroscopic debridement without subacromial decompression seems to be advisable for patients with therapy-resistant calcifying tendinitis of the shoulder.

*Level of evidence *2, Open-Label Randomized Clinical Trial.

*IRB* METC Zuyderland MC. Number: 14-T-112.

*Registered at trialregister.nl* NL 4947.

## Introduction

Calcifying tendinitis of the shoulder is a common shoulder disease that often leads to long-lasting pain and loss of function [1–3]. The supraspinatus tendon is the most affected rotator cuff tendon [1, 2]. The exact etiology of calcifying tendinitis of the shoulder remains unclear [3–6]. The initial treatment of calcifying tendinitis of the shoulder is with conservative or minimal invasive measures (such as shock wave therapy or needle aspiration of calcific deposit (NACD). However, about 10% of the patients require surgical treatment [6–9]. Several surgical treatment options are shown to be effective [10, 11]. However, the most effective surgical treatment procedure has not been appointed yet [10]. In general, there are three surgical treatment options available. The first is to perform an arthroscopic subacromial decompression without debridement of the calcifications [12–14]. The second option is to perform an arthroscopic debridement of the calcifications without subacromial decompression, and the third option is to perform an arthroscopic debridement of the calcifications combined with subacromial decompression. It is not known which of these surgical treatment procedures is the most effective concerning short-term pain relief and functional outcomes [10, 11, 15, 16].

Therefore, the purpose was to compare these three surgical treatment procedures regarding pain relief and functional outcomes 6 months after surgical treatment. We hypothesized that all three procedures have comparable effectiveness concerning pain relief and functional outcome.

## Materials and methods

After approval of the medical ethics review committee (METC) of both centers (number: 14-T-112) and registration in the Dutch clinical trial registry (number: NL 4974), an open-label dual-center randomized clinical trial was conducted between September 2015 and June 2020. Patients were randomly assigned by computer into three treatment groups: 1. arthroscopic subacromial decompression without debridement of the calcifications (Group SAD), 2. arthroscopic debridement of the calcifications without subacromial decompression (Group D), and 3. arthroscopic debridement of the calcifications combined with subacromial decompression (Group D + SAD).

### Patient eligibility

**Fig. 1:**
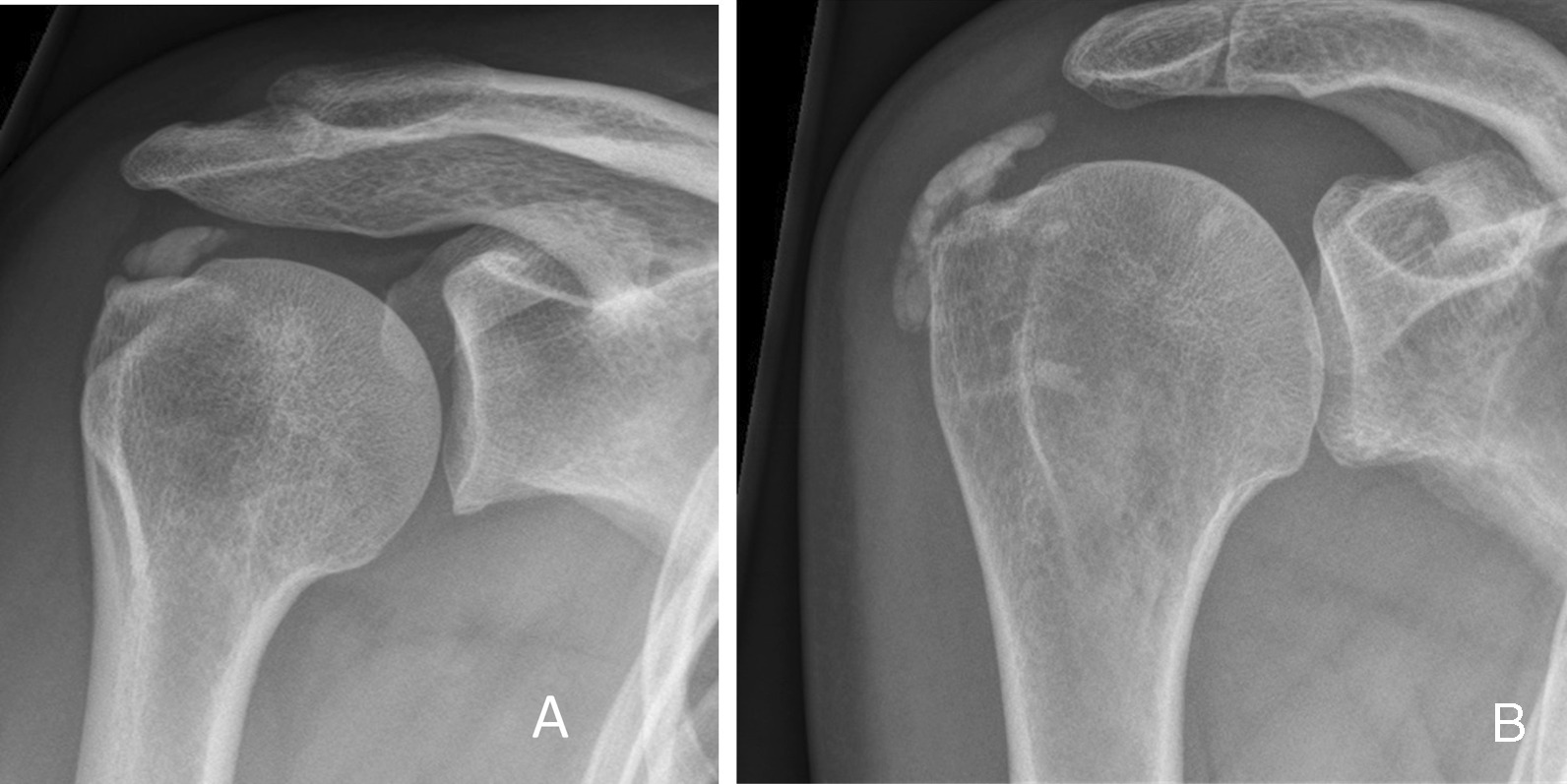
Gartner type I and II calcifications. **A** Clearly circumscribed, dens subacromial calcification (Gartner type I) on shoulder AP view; **B** partially clearly circumscribed, partially heterogeneous, partially dens subacromial calcification (Gartner type II) on shoulder AP view

All patients were recruited by experienced orthopedic shoulder surgeons in two different hospitals (SK and EJ in Zuyderland MC and OLH in Viecuri MC). Patients with a prolonged course of calcifying tendinitis of the shoulder in which surgical treatment was chosen as a treatment were considered for eligibility. Inclusion criteria were failed conservative treatment (including at least a subacromial injection (SAI) in combination with exercise therapy and/or nonsteroidal anti-inflammatory drugs (NSAIDs) for at least 6 months, type I or II subacromial calcifications according to the Gartner classification[4] (Fig. 1), calcifications with diameter more than 5 mm (Bosworth Grade 2–3) [17], unrestricted range of motion (> 150° abduction/anteflexion and > 70° external rotation) and able and willing to participate in the study. Exclusion criteria were signs of adhesive capsulitis, symptomatic degenerative diseases of the acromioclavicular joint, full-thickness rotator cuff lesions, fibromyalgia, rheumatoid arthritis, surgical history of the affected shoulder, perioperative findings of significant intraarticular pathology (e.g., biceps pathology or acromioclavicular/glenohumeral osteoarthritis), and Gartner type III subacromial calcifications.

### Operative technique and rehabilitation protocol

**Fig. 2:**
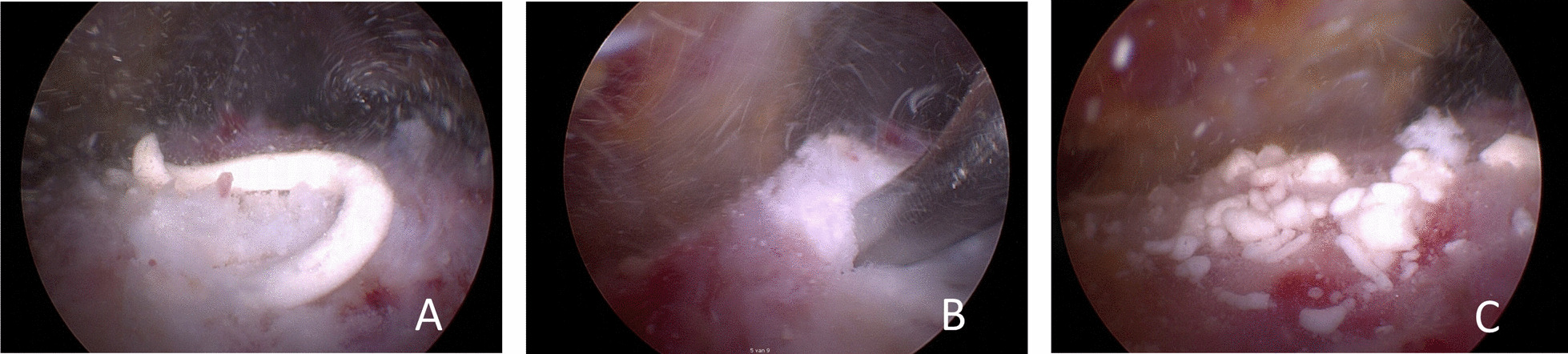
Arthroscopic debridement of calcifications. **A** After needling with 18-gauge needle, a toothpaste-like calcification discharges; **B** after a small bursal side in line with the tendon fibers incision and calcific deposit is arthroscopically debrided by applying pressure using a curette and a shaver; **C** after debridement of the calcification the subacromial space is extensively rinsed to eliminate as much calcific deposit particles as possible

**Fig. 3:**
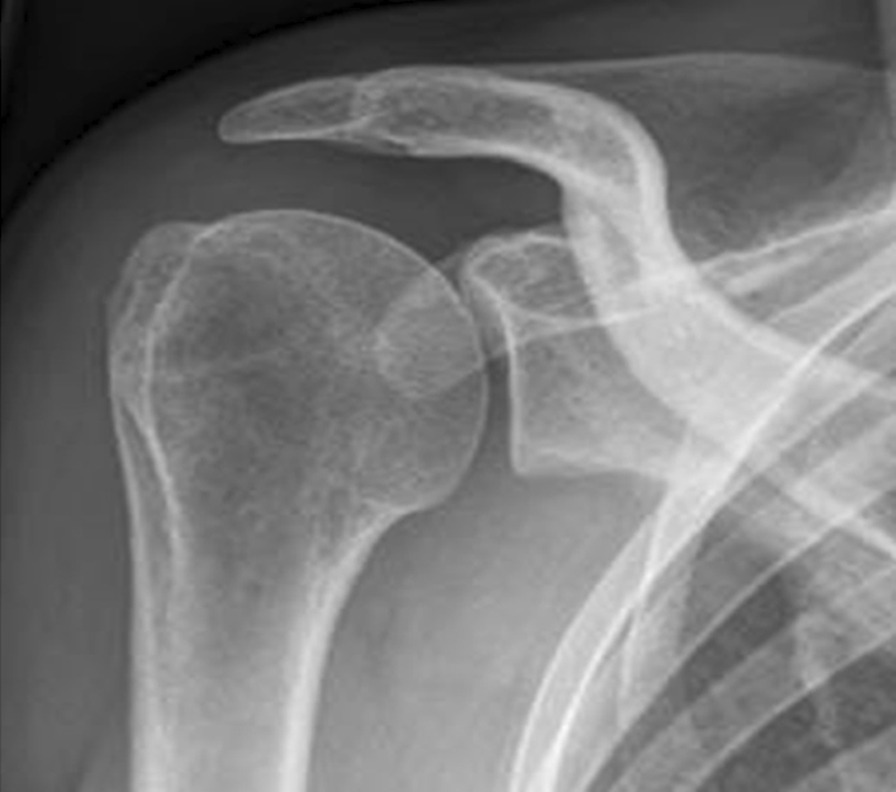
Postoperative status after subacromial decompression

All surgical procedures were performed by experienced shoulder surgeons (EJP, OLH, SK). After general and/or regional anesthesia a standardized diagnostic glenohumeral arthroscopy was performed to assess possible intraarticular and full-thickness rotator cuff lesions. Then a subacromial bursectomy was performed. In patients allocated to Group D and Group D + SAD, the calcification was located using an 18-gauge needling technique described by Ellman [18]. A small bursal-sided partial-thickness rotator cuff defect was made (Ellman grade 1–2) in line with the tendon fibers and a calcific deposit was arthroscopically debrided by applying pressure using a curette and shaver (Fig. 2) [18]. Rotator cuff repair was not deemed necessary in any of the patients. In patients allocated to Group SAD and D + SAD, an arthroscopic SAD was performed as described by Caspari and Thal (Fig. 3) [19]. The rehabilitation protocol was the same in all groups and consisted of a sling for several days, pain-based movements and NSAIDs as necessary for 2 weeks.

### Outcome measures

Patients were invited for clinical and radiological evaluation at the orthopedic outpatient department 6 weeks and 6 months after surgery.

### Primary outcome

#### Visual analog scale

The primary outcome was the difference in an improvement in the visual analog scale for pain (pVAS) 6 months after surgical treatment between the three groups. The pVAS was recorded at the start of the study, 6 weeks and 6 months after surgery [20].

### Secondary outcomes

#### Functional outcome

The functional outcome was assessed preoperatively, 6 weeks and 6 months after surgery using Constant-Murley Score (CMS) [21], Disability of Arm, Shoulder and Hand score (DASH) [22], and American Shoulder and Elbow Surgeons score (ASES) [23].

#### Radiological outcome

Preoperatively, 6 weeks and 6 months postoperatively standardized radiographs were taken. Radiological evaluation was done by one author (FV) and consisted of an evaluation of plain radiographs taken in three directions: true anteroposterior view, anteroposterior view with arm in internal rotation, and anteroposterior view with arm in external rotation. Using these radiographs, calcifications were classified using Gartner classification [4] and Bosworth classification [17]. Furthermore, the size and location of the calcifications were measured. Results of additional imaging techniques (e.g., MRI or sonography) were retrieved from electronic patient files. Six weeks and 6 months postoperatively, the size of residual calcification was measured by the same author (FV). In the case of multifocal or heterogeneous calcifications, the size was added up and presented as the sum of the sizes of the calcifications.

#### Additional treatments and complications

Complications such as postoperative adhesive capsulitis and infection were registered. Furthermore, any protocol violations such as additional treatments (e.g., subacromial injections and number of needle aspirations of calcific deposit procedures) were recorded. The decision to perform additional treatments was made after deliberation of the treating physician with the research team and the patient. It was documented and retrieved from the electronic patient files. When the decision was made to perform an additional treatment, adverse events such as rotator cuff lesions (confirmed on ultrasound or MRI) and adhesive capsulitis were first ruled out. Failure to treatment was defined as a decrease in VAS of less than 20 mm 6 months postoperatively [24].

### Sample size calculation and power analysis

The study was designed using an equivalence model. The trial was powered to detect a difference of 15 mm on the pVAS between the three treatment groups, since 15 mm was the minimal clinically important difference in patients after rotator cuff surgery in the study of Kim et al. [24]. The sample size calculation estimated that we needed to include 34 patients per group using an alpha of 0.05. This would provide a power of 90%. Considering that 10% of patients would be lost to follow-up, the study population needed to consist of 114 patients.

### Statistical analyses

The data were analyzed using SPSS version 26.0 (IBM Corp., Armonk, NY). The data were analyzed using an intention-to-treat analysis (ITT analysis). Furthermore, a per-protocol analysis (PP analysis) was performed. Data of the ITT analysis are presented. In the cases of statistically significant differences between the ITT analysis and PP analysis, data for both analyses are presented. Normally distributed data are presented as a mean with standard deviation. Non-normally distributed data are presented as a median and range. Differences in baseline characteristics were analyzed with a one-way ANOVA test for continuous variables and chi-square test for categorical variables for normally distributed data. Non-normally distributed data were analyzed with a Kruskal–Wallis one-way ANOVA for non-normally distributed data. The outcomes were analyzed using a one-way ANOVA (with a Tukey’s adjustment for multiple testing). *p* ≤ 0.05 was considered statistically significant for all tests.

## Results

### Study population

**Fig. 4:**
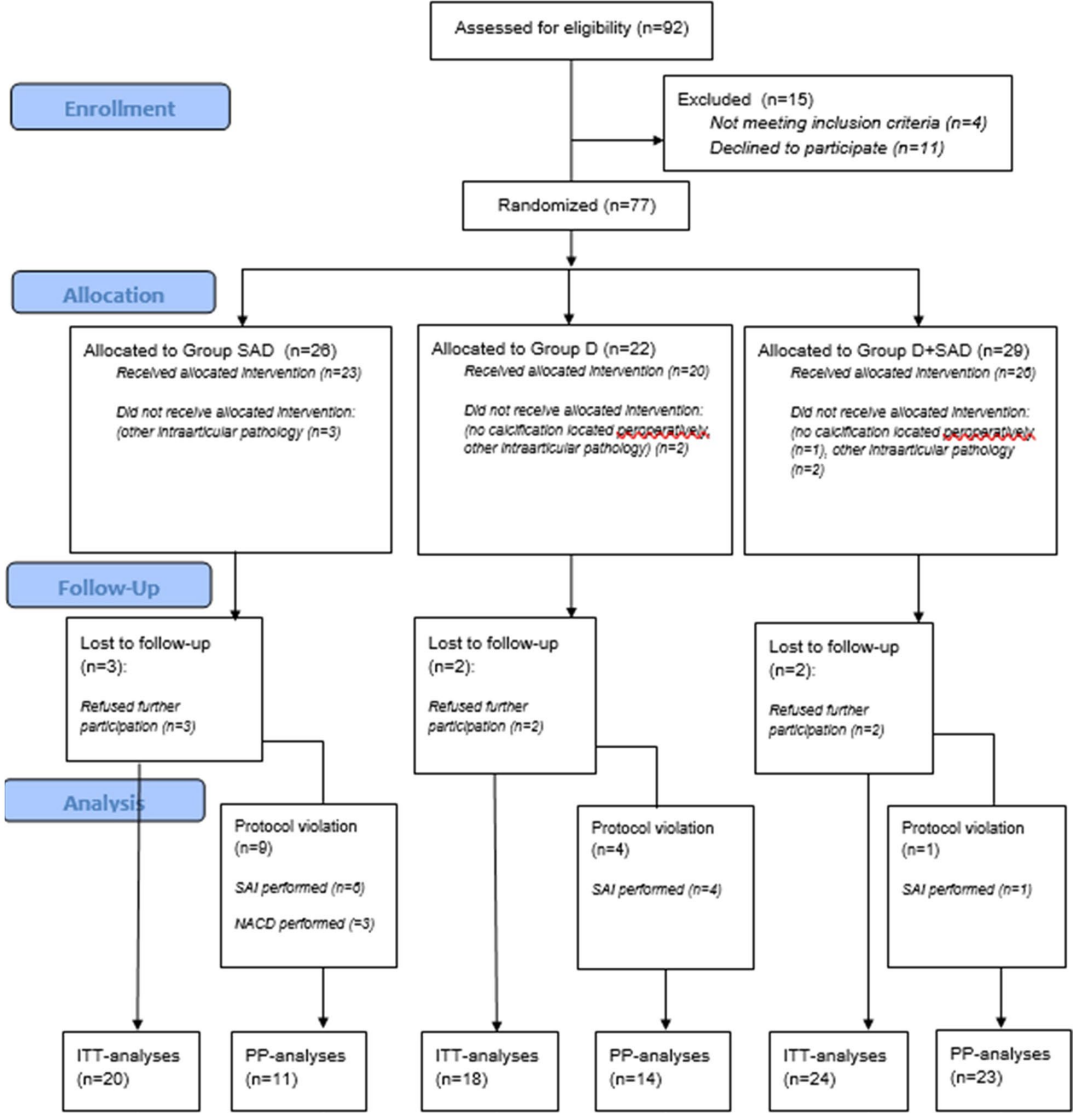
CONSORT Flowchart. *Group SAD* arthroscopic subacromial decompression, *Group D* arthroscopic debridement, *Group D + SAD* arthroscopic subacromial decompression and debridement. *NACD* needle aspiration of the calcific deposit, *SAI* subacromial injection, *ITT analysis* intention-to-treat analysis, *PP analysis* per-protocol analysis

As can be seen in the CONSORT Flowchart, 77 patients were considered eligible and were randomized into three treatment groups (Fig. 4). Four patients were excluded because of significant other intraarticular pathology (e.g., two patients with significant acromioclavicular osteoarthritis and two patients with biceps pathology). Two patients were excluded from further analyses because the calcification could not be located at the time of the intervention and thus could not be debrided. These two patients received subacromial decompression without debridement of the calcifications. The mean follow-up was 6.4 months (SD, 1.3 months). The overall follow-up rate after 6 weeks and 6 months was 97.1% and 89.9%, respectively. Thus, seven patients were lost to follow-up. These patients were contacted by phone and stated they did not receive other treatments for the affected shoulder. The baseline characteristics are given in Table 1.

**Table 1:** Baseline characteristics

	Complete study population (*n* = 62)	Group SAD (*n* = 20)	Group D (*n* = 18)	Group D + SAD (*n* = 24)	*p* value
Age in years (SD)	53.5 (9.0)	52.7 (10.1)	54.9 (7.7)	53.2 (8.8)	0.82
Gender male (%)	21 (33.9)	7 (35.0)	3 (16.7)	11 (45.8)	0.13^*^
Affected side Right (%)	47 (75.8)	15 (75.0)	16 (88.9)	16 (66.7)	0.24^*^
Dominant side Right (%)	46 (74.2)	14 (70.0)	16 (88.9)	16 (66.7)	0.20^*^
Smoking status smoking (%)	18 (29.0)	6 (20.0)	3 (16.7)	9 (37.5)	0.48^*^
ASA category					
1 (%)	26 (41.9)	8 (40.0)	8 (44.4)	10 (41.7)	0.99^*^
2 (%)	32 (51.6)	10 (50.0)	11 (61.1)	11 (45.8)	
3 (%)	4 (6.5)	1 (5.0)	1 (5.6)	2 (8.3)	
BMI Mean (SD)	26.7 (4.3)	26.4 (4.5)	26 (3.5)	27.3 (4.7)	0.78
Comorbidities					
Pulmonary (%)	12 (19.4)	4 (20.0)	1 (5.6)	7 (29.2)	0.21
Cardiovascular (e.g., HT, hyperlipidemia)	9 (14.5)	2 (10.0)	3 (16.7)	4 (16.7)	0.91
Thyroid dysfunction	2 (3.2)	0	1 (5.6)	1 (4.2)	NA
Diabetes mellitus	1 (1.6)	0	0	1 (4.2)	NA
Duration of symptoms in months (range)	22.2 (6–120)	17.1 (6–120)	20.3 (6–120)	24.7 (6–120)	0.57^+^
Size of deposit in mm	23.0 (7.6)	23.4 (6.0)	24.7 (7.3)	21.3 (8.6)	0.34
Bosworth grade					
II, medium (%)	5 (8.1)	1 (5)	0	4 (16.7)	0.34
III, large (%)	57 (91.9)	19 (95)	18 (100.0)	20 (83.3)	
Gartner type					
Type I (%)	39 (62.9)	15 (75.0)	11 (61.1)	13 (54.2)	0.62^*^
Type II (%)	23 (37.1)	5 (25.0)	7 (38.9)	11 (45.8)	
Location of deposit					
SSP (%)	54 (87.1)	17 (85.0)	16 (88.9)	21 (87.5)	0.87^*^
ISP (%)	8 (12.9)	3 (15.0)	2 (11.1)	3 (12.5)	
SSC (%)	0 (0.0)	0 (0.0)	0 (0.0)	0 (0.0)	

*SAD* subacromial decompression, *D* debridement of calcifications, *D + SAD* debridement and subacromial decompression; *, x^2^ test; ^+^, Kruskal–Wallis test; *ASA* American Society of Anesthesiologists Physical Status, *BMI* Body Mass Index, *HT* hypertension, *NA* not applicable, *SSP* supraspinatus, *ISP* infraspinatus, *SSC* subscapularis; (_), standard deviation

Baseline characteristics did not differ statistically significantly between groups. In 52 patients (83.9%), preoperative additional imaging tests were performed. In 50 patients (80.6%), an ultrasound was performed and two patients had an additional MRI scan. No partial or full-thickness rotator cuff lesions were observed preoperatively. Data from the ITT analyses are presented since no significant differences were apparent between the ITT analysis and the PP analyses.

### Primary outcome

#### Visual analog scale after 6 months

Patients in all groups showed a statistically significant improvement in pVAS 6 months after surgical intervention (*p* < 0.001), and it did not differ statistically significantly between groups (Table 2).

**Table 2:** Outcome score after surgery

	Group SAD	Group D	Group D + SAD	*p* value
VAS for pain				
Baseline	58.8 (15.3)	61.6 (16.3)	58.1 (13.7)	0.74
6 weeks	42.3 (17.0)	30.6 (16.8)	25.1 (19.0)	0.03*
6 months	17.0 (16.0)	12.2 (11.7)	11.4 (14.6)	0.57
CMS				
Baseline	43.0 (13.9)	45.1 (10.3)	51.0 (12.9)	0.10
6 weeks	66.3 (18.0)	72.1 (22.8)	79.3 (16.0)	0.77
6 months	84.8 (18.0)	89.7 (9.6)	91.8 (11.2)	0.84
DASH				
Baseline	49.2 (21.9)	45.0 (21.1)	45.2 (13.6)	0.80
6 weeks	37.6 (20.8)	24.9 (16.5)	14.7 (13.7)	0.02*
6 months	16.3 (14.1)	10.1 (11.5)	8.2 (14.4)	0.89
ASES				
Baseline	41.5 (18.0)	40.4 (12.9)	44.8 (12.5)	0.63
6 weeks	62.5 (21.2)	71.9 (16.2)	77.1 (18.7)	0.21
6 months	83.2 (15.0)	88.9 (10.7)	85.9 (19.3)	0.50
Size of calcification				
Baseline	23.4 (6.0)	24.7 (7.3)	21.3 (8.6)	0.34
6 weeks	14.2 (9.4)	10.3 (7.6)	8.1 (6.1)	0.02*
6 months	6.1 (8.9)	1.0 (3.0)	1.0 (2.7)	0.03*

*SAD* subacromial decompression, *D* debridement of calcifications; *D + SAD* debridement and subacromial decompression, *VAS* mean visual analog scale, *CMS* mean Constant-Murley score, *DASH* mean disability of arm, shoulder and hand score, *ASES* mean American shoulder and elbow surgeons score;*, statistically significant (e.g., *p* < 0.05) between Group D + SAD and SAD; (_), standard deviation

### Secondary outcomes

#### Visual analog scale after 6 weeks

**Fig. 5:**
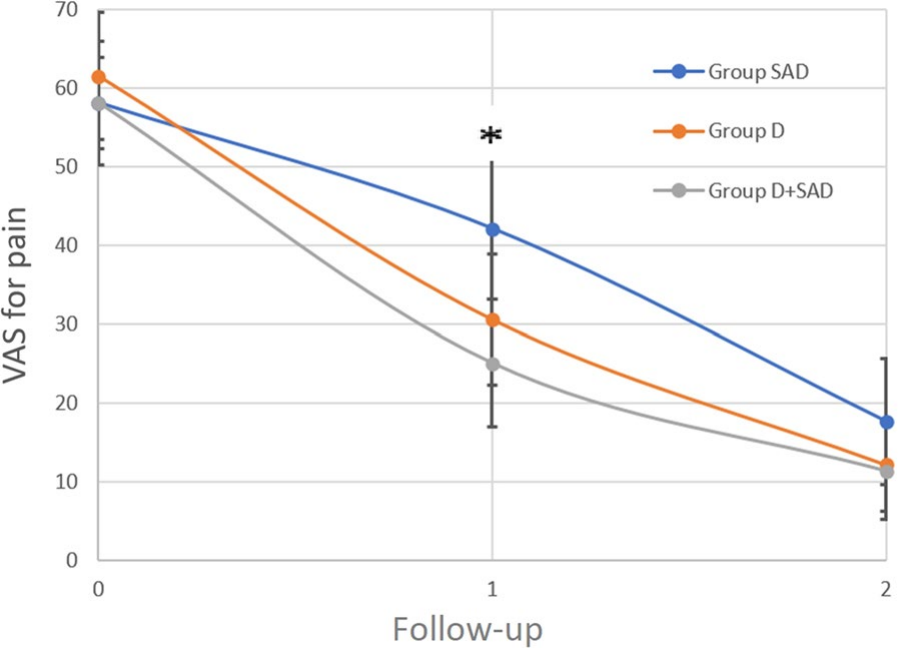
Estimates of pain scores (measured with pVAS) during the follow-up. *SAD* subacromial decompression, *D* debridement of calcifications, *D + SAD* debridement and subacromial decompression, *VAS* mean visual analog scale; follow-up 1, 6 weeks; follow-up 2, 6 months; *, statistically significant differences (e.g., *p* < 0.05) between Group D + SAD and SAD

An improvement in pVAS was significantly more (*p* = 0.03) in Group D + SAD (33.1 mm; SD, 19.7 mm) compared to Group SAD (16.5 mm; SD, 19.3 mm) (Fig. 5). Six weeks and 6 months after treatment, no statistically significant differences between Group D and Group D + SAD were observed.

#### Functional outcome

In Table 2, the functional outcome scores are summarized. In all three groups, the CMS improved significantly (*p* < 0.0001). Six weeks and 6 months after treatment, no statistically significant differences between the groups were observed.

**Fig. 6:**
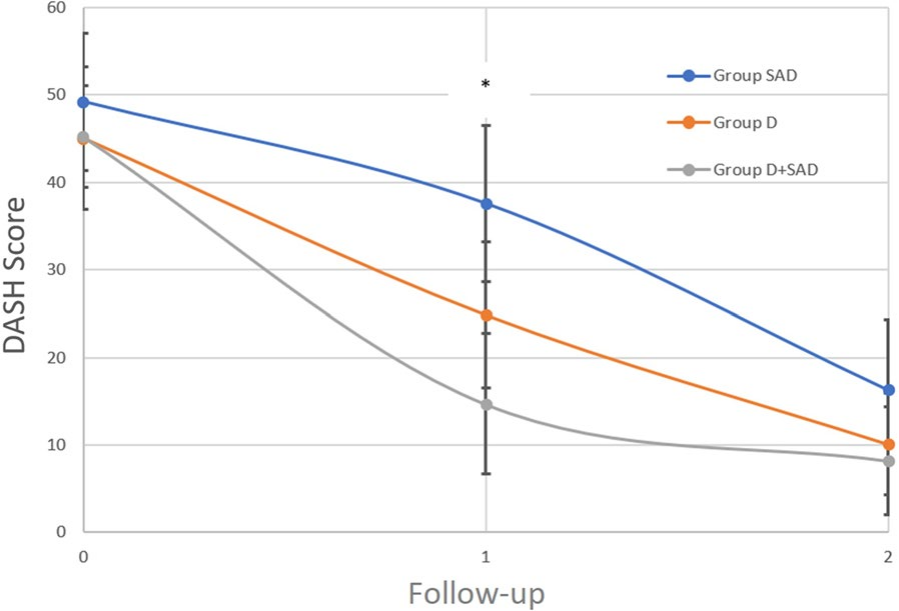
DASH score during follow-up. *SAD* subacromial decompression, *D* debridement of calcifications, *D + SAD* debridement and subacromial decompression, *VAS* mean visual analog scale; follow-up 1, 6 weeks; follow-up 2, 6 months; *, statistically significant differences (e.g., *p* < 0.05) between Group D + SAD and Group SAD

The DASH score in all patient groups decreased statistically significantly during the study period (*p* < 0.0001). Six months after treatment, no statistically significant differences between the groups were observed (Fig. 6). At 6 weeks postoperatively, the improvement in DASH score was significantly higher (*p* = 0.02) in Group D + SAD (30.6; SD 17.8) compared to Group SAD (11.6; SD 24.3). Six weeks and 6 months after treatment, no statistically significant differences between Group D and Group D + SAD were observed.

In all three groups, ASES improved significantly (*p* < 0.0001). Six weeks and 6 months after treatment, no statistically significant differences between the groups were observed.

#### Radiological outcome

**Fig. 7:**
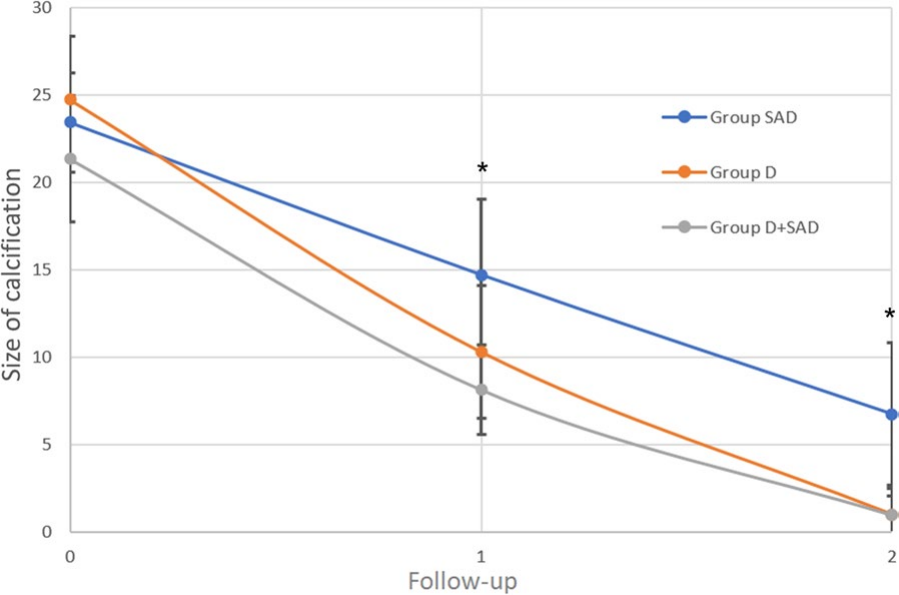
Size of calcification during follow-up. *SAD* subacromial decompression, *D* debridement of calcifications, *D + SAD* debridement and subacromial decompression; follow-up 1, 6 weeks; follow-up 2, 6 months additional treatments and complications

All three groups showed a statistically significant (*p* < 0.0001) decrease in the size of the calcification from 23.1 mm (SD, 7.6 mm) to 2.3 mm (SD, 5.6 mm) at the 6 months of evaluation (Fig. 7). The decrease was statistically significant less (*p* = 0.04) in Group SAD compared to Group D after 6 weeks and 6 months. The difference in the decrease in the calcification between Group SAD and Group D + SAD was near significant (*p* = 0.06) after 6 months and significant after 6 weeks (*p* = 0.04). Six weeks and 6 months after treatment no statistically significant differences between Group D and Group D + SAD were observed. The clinical outcome did not differ significantly between patients with and without the presence of any residual subacromial calcifications (VAS for pain, *p* = 0.96; CMS, *p* = 0.82; DASH, *p* = 0.64; ASES, *p* = 0.94).

In Table 3, the additional treatments and complications are summarized. No reoperations were performed, and no full-thickness rotator cuff lesions were documented during the follow-up.

**Table 3:** Additional treatments and complications during follow-up

	Group SAD	Group D	Group D + SAD	*p* value
Additional treatments	9 (45)	4 (22.2)	1 (4.2)	0.004^*^
NACD	3 (15)	0 (0.0)	0 (0.0)	
SAI	6 (30)	4 (22.2)	1 (4.2	
Complications	2 (10)	1 (5.5)	1 (4.2)	0.73
Treatment failure	3 (15)	1 (5.5)	4 (16.8)	0.54

*SAD* subacromial decompression, *D* debridement of calcifications, *D + SAD* debridement and subacromial decompression, *NACD* needle aspiration of calcific deposit, *SAI* subacromial injection; (_), % of patient group; *, statistically significant differences (e.g., *p* < 0.05) between Group D + SAD and SAD

In Group SAD, 45.0% (*n* = 9) received additional treatments during follow-up: three patients received an additional NACD because of unchanged symptoms of pain and impaired shoulder function between four and 6 months after the initial treatment. Six patients received a subacromial injection with lidocaine and a corticosteroid (SAI). This was significantly more compared to Group D + SAD (*p* = 0.003). The complication rate in Group SAD was 10.0%. Two patients (10%) developed an adhesive capsulitis. This did not differ significantly between the three treatment groups (*p* = 0.73). Three patients (15.0%) showed pain relief less than 20 mm and were therefore classified as a failure to treatment. This did not differ significantly between the three treatment groups (*p* = 0.54).

In Group D, 22.2% (*n* = 4) received additional treatments during follow-up: four patients received a SAI 4 to 6 months after surgery due to persisting pain. The complication rate in Group D was 5.5%. One patient developed an adhesive capsulitis two months after treatment, which was resolved at the final follow-up.

In Group D + SAD, 4.2% (*n* = 1) received additional treatments during follow-up: one patient received a SAI 3 months after surgery. The complication rate in Group D + SAD was 4.2%. One patient developed an adhesive capsulitis 3 months after treatment, which was resolved at final follow-up.

#### Post hoc sample size calculation and power analysis

An interim analysis was performed after the recruitment came to a halt due to the COVID-19 pandemic. With the results of the interim analysis, actual standard deviations of the primary outcome (21.2 mm) were calculated. The post hoc sample size calculation showed that we would need to include 121 patients per group using an alpha of 0.05. This would provide a power of 80%. This was not deemed feasible, and after deliberation among the authors and the local ethical board the study was stopped before the required sample size was achieved at a number of 77 patients (67.5%).

## Discussion

This is the first multicenter randomized clinical trial that compares three surgical treatment procedures for therapy-resistant calcifying tendinitis of the shoulder. In this study, all patient groups showed significant pain relief and improvement in shoulder function 6 months after surgery. However, patients who had a SAD showed inferior improvement in pVAS and DASH score after 6 weeks. Furthermore, this group required more postoperative additional treatments. No significant differences were observed between patients in Group D and D + SAD. Therefore, an arthroscopic debridement without subacromial decompression seems to be advisable.

Whether or not to debride the calcifications is still a matter of dispute [12–14]. The present study is the first to compare the short-term effectiveness of these procedures in a randomized clinical trial. Previous studies on this topic have a retrospective or a non-randomized design [10]. Hofstee [12] reported results of a pseudo-randomized study. No differences in the functional outcome were observed between patients who had a SAD compared to patients who had a D + SAD after a mean follow-up of 36 months. Furthermore, Tillander et al. [13] reported a cohort study in which patients were reviewed 24 months after SAD alone. The two groups in this study were patients with calcifying and non-calcifying tendinitis of the rotator cuff and no differences were apparent in the functional outcome. In both studies, complete disappearance or significant decrease in size of the calcifications was frequently observed [12, 13, 25]. Therefore, it can be hypothesized that the surgical procedure with the SAD serves as a stimulus for the tendon to return to the resorption phase. This resorption phase is known to cause symptoms of pain and impaired function [26]. Even a chemical subacromial bursitis can occur caused by erupting hydroxyapatite crystals [27]. In the present study, calcifications disappeared in almost half of the patients during the follow-up. This aforementioned resorption process might explain why significantly more postoperative additional treatments were necessary for patients in Group SAD. Hence, these additional treatments (e.g., SAIs and NACDs) were performed between four to 6 months after the initial surgical treatment. One can hypothesize that the actual clinical outcomes are worse in patients who had a SAD as they were influenced by these additional treatments. Therefore, based on the finding that more additional treatments were necessary and worse pVAS and DASH scores when the calcifications were left untouched it seems that debridement of the calcifications is preferential.

In the present study, a SAD did not seem to be beneficial in addition to debridement of the calcifications. After a mean follow-up of 6 months, the primary outcome and all secondary outcomes did not differ between patients in Group D compared to D + SAD. These findings are in line with the randomized controlled study of Clement [11]. They found that after a mean follow-up of 12 months pain relief and improvement in shoulder function were not influenced by the additional SAD. This is in contradiction with the study of Balke [15]. In this study, patients received an additional SAD if perioperative signs of subacromial impingement were observed. Patients who received an additional SAD showed superior scores on the ‘pain’ component of both the CMS and the ASES scores [15]. Though the difference was small, the clinical relevance can be questioned since it did not exceed the minimal clinical difference (MCID). Furthermore, in contrast to our study these patients were not randomized for an additional SAD and therefore selection bias could have occurred. In the present randomized clinical trial, no between-group differences were observed between patients in Group D and D + SAD. However, in Group D + SAD four patients failed to have satisfactory pain relief at the final follow-up compared to an only patient in Group D. It can be hypothesized that the recovery pattern is more prolonged by performing an additional subacromial decompression because it is a more extensive procedure. These findings are in line with the study of Cho et al. [28] Therefore, based on these short-term findings an additional subacromial decompression does not seem to be beneficial.

### Limitations

Some limitations apply to the current study. Firstly, since the study was terminated prematurely it could be underpowered, which could lead to sampling bias and underestimation of the between-group differences. This should be considered when interpreting the results. Secondly, the lack of a control group in which a sham operation or no surgical treatment was performed. This was left out because of ethical considerations. The aim of this study was to compare the short-term effectiveness of three surgical treatment procedures and not to assess the efficacy of surgery. Several previous studies have been published that assure that a surgical treatment is a safe and effective treatment option with good short- and midterm radiological and clinical outcomes [9, 10, 29, 30]. Thirdly, a follow-up of 6 months can be criticized for being too short since the recovery period after shoulder surgery can be prolonged. However, in the study of Yoo et al. [31] 85.7% of the patients’ maximal pain relief was achieved within 6 months after surgery [31]. It was assumed that if differences in the effectiveness between treatment groups would be present they most likely could be detected in the short term. Moreover, with this short-term follow-up the impact of the natural course of calcifying tendinitis of the shoulder on the outcome scores was reduced. Finally, the lack of control group in which complete debridement of the calcifications with additional rotator cuff repair was performed which is advocated by some authors [31, 32]. However, an additional rotator cuff repair is also reported to be a negative prognostic factor and a frequently seen complication is adhesive capsulitis with reported incidences as high as 29% [28, 31]. Postoperative immobilization after rotator cuff repair is a known risk factor for the development of adhesive capsulitis [31]. Therefore, the investigated surgical procedures were favored by the authors. This made rehabilitation possible with the usage of a sling for a few days and immediate pain-based movements to decrease the likelihood of an adhesive capsulitis. This surgical technique is supported by other studies [12, 26, 28, 31].

## Conclusions

All patient groups showed significant pain relief and an improvement in shoulder function 6 months after surgery. However, patients in Group SAD showed inferior pain relief and an improvement in DASH score after 6 weeks. Furthermore, this group required more postoperative additional treatments. No significant differences in clinical and radiological outcomes were observed between patients in Group D compared to Group D + SAD. Therefore, an arthroscopic debridement without subacromial decompression seems to be advisable for patients with therapy-resistant calcifying tendinitis of the shoulder.
